# Experiments with LDA and Top2Vec for embedded topic discovery on social media data—A case study of cystic fibrosis

**DOI:** 10.3389/frai.2022.948313

**Published:** 2022-08-18

**Authors:** Bradley Karas, Sue Qu, Yanji Xu, Qian Zhu

**Affiliations:** ^1^Division of Rare Diseases Research Innovation, National Center for Advancing Translational Sciences (NCATS), National Institutes of Health (NIH), Bethesda, MD, United States; ^2^Division of Pre-Clinical Innovation, National Center for Advancing Translational Sciences, (NCATS), National Institutes of Health (NIH), Rockville, MD, United States

**Keywords:** rare disease, cystic fibrosis, Reddit, topic modeling, LDA, Top2vec

## Abstract

Social media has become an important resource for discussing, sharing, and seeking information pertinent to rare diseases by patients and their families, given the low prevalence in the extraordinarily sparse populations. In our previous study, we identified prevalent topics from Reddit via topic modeling for cystic fibrosis (CF). While we were able to derive/access concerns/needs/questions of patients with CF, we observed challenges and issues with the traditional techniques of topic modeling, e.g., Latent Dirichlet Allocation (LDA), for fulfilling the task of topic extraction. Thus, here we present our experiments to extend the previous study with an aim of improving the performance of topic modeling, by experimenting with LDA model optimization and examination of the Top2Vec model with different embedding models. With the demonstrated results with higher coherence and qualitatively higher human readability of derived topics, we implemented the Top2Vec model with doc2vec as the embedding model as our final model to extract topics from a subreddit of CF (“r/CysticFibrosis”) and proposed to expand its use with other types of social media data for other rare diseases for better assessing patients' needs with social media data.

## Introduction

The call for “recognizing the need to promote and protect the human rights of all persons that included the estimated 300 million persons living with a rare disease worldwide” was made with UN Resolution in 2021^1^ which was adopted by all 193 UN Member States. This resolution no only aimed to raise awareness and promote advocacy for rare disease research but also addressed the challenges of those rare disease patients and their families. In addition, in January 2019, the US Food and Drug Administration (FDA) published a revised draft guidance on rare and orphan drug development (U.F.a.Administration, 2019). “The guidance encourages researchers to involve patients, caregivers, and advocates and having them provide input on their experiences, perspectives, and priorities related to potential end points used during the drug-development process and regulatory review. The guidance also encourages the use of social media as a means to represent the perspective of the patients” (Merinopoulou and Cox, 2019). Clearly, analyzing social media data allows effectively accessing challenges of rare diseases from patients' perspectives, since social media and online social networks aim to connect people all around the world. Rare disease patients and caregivers are often geographically dispersed and isolated, making it difficult for them to communicate with others about their conditions. Thus, there is an increasing number of patients and caregivers turning to social media for seeking information related to their diseases.

Social media has been increasingly applied in biomedical research, as Lim et al. outlined and discussed the opportunities of using social media in medical and health care (Lim, 2016). Many studies applied social media for not only disease management, surveillance, and trend prediction in chronic diseases (Reich et al., 2019; Abouzahra and Tan, 2021; Madhumathi et al., 2021), but also for rare disease applications (Mallett et al., 2019; Choudhury et al., 2021). Among those applications, natural language processing (NLP) as a main computational approach has been employed to analyze social media in free text. Sarker et al. (2022) analyzed posts from 14 opioid-related forums on the social network, Reddit using NLP, and compared concerns to treatment and access to care among people who use opioids before and during the COVID-19 pandemic. Furthermore, topic modeling, as an important NLP method, has been widely applied to identify hidden topics from social media to support biomedical research. Using Reddit data, researchers investigated people's concerns about the human papillomavirus vaccine (Lama et al., 2019), the discourse about people using cannabis, tobacco, and vaping (Benson et al., 2021), discussion topics of online depression community (Feldhege et al., 2020), and topics of persons with emotional eating behavior (Hwang et al., 2020), as well as public sentiment on COVID-19 vaccines (Melton et al., 2021). These studies applied Latent Dirichlet Allocation (LDA) (Blei et al., 2003) for topic modeling.

Latent Dirichlet Allocation is an unsupervised, probabilistic modeling method, which extracts topics from a collection of documents. Each topic is made up of the probabilistic distribution of words contained in that topic. The words that have the highest probability for that topic are used to describe the contents of that topic. These probability distributions of words are used to assign probabilities of topics to each document. Limitation of LDA on topic modeling has been reported that included the need for data cleaning and pre-processing, selection of model parameters, such as the number of topics, and interpretability and validation of the generated topics. (Maier et al., 2018; Angelov, 2020) Consequently, more advanced algorithms have been invented as an alternative technique for topic modeling, such as Top2Vec. (Angelov, 2020) Top2Vec is an algorithm that takes a collection of input texts and converts each word in the text to a vector in semantic space using an encoding model, such as doc2vec. With that, it can automatically detect topics present in the text and generate jointly embedded topic, document, and word vectors. There are studies that compared LDA and Top2Vec (Ma et al., 2021; Egger and Yu, 2022). They reported that Top2Vec produced qualitatively higher quality results than LDA. However, they did not apply any quantitative methods for comparing derived topics from different algorithms. Instead, in this study, we compared their performance via a quantitative method for analyzing topic model results using different methods.

Cystic fibrosis (CF) is a genetic (inherited) disease that affects multiple organs in the body (L. National Heart, and Blood Institute, 2021). CF results in the accumulation of mucous in various cells and tissues, which leads to persistent lung infections, nutritional problems, and other serious manifestations. CF is one of the more common genetic disorders in the United States, occurring in one of every 3,200 live births and affecting about 30,000 people in the US^2^^,^^3^ In this study, we analyzed posts and comments relevant to CF from Reddit, to examine and compare the performance of LDA and Top2Vec for hidden topic extraction, in order to better understand patients' needs from the patients and/or their families' perspective.

## Materials and methods

Given those limitations with the LDA model that we observed from our previous study (Zhu et al., 2021), in this study, we aimed at discovering solutions to accurately identify prevalent topics from Reddit for CF. Here, we described several experiments we performed accordingly, such as tuning different parameters to optimize the LDA model for better performance and validating the performance of the newly invented Top2Vec model, for topic modeling.

### Previous study

In our previous study (Zhu et al., 2021), we implemented an LDA model (Blei et al., 2003; Hoffman et al., 2010) with the Gensim Python package (Rehurek and Sojka, 2010) to derive five main topics, i.e., Daily Life, Medication/Symptom, Testing/Diagnosis, Insurance, and Medical Equipment, from a CF-related Reddit discussion community. Discussion threads on Reddit are organized as subreddits, the subreddit of “r/CysticFibrosis” is a discussion forum for CF. By using LDA, there is no direct means to determine the representative number of topics, and since LDA is based on the bag of word strategy, no semantics among words presented in the text have been taken into consideration. This results in unrelated words that might be used and contributing to generate those topics, which leads to difficulty for humans to understand the meaning of a topic. Given those limitations observed, corresponding extensions are presented here, (1) automatically detecting topics from the text and (2) extracting topics with high precision. Experiments have been conducted accordingly to compare LDA and Top2Vec and are described in the below sections.

### Rare disease data preparation

#### Data collection

In this study, we collected posts and their associated comments if applicable from the subreddit of “r/CysticFibrosis” using the Programming Models and Algorithms Workshop (PMAW)^4^, which is a multithreaded wrapper for the Pushshift API (Baumgartner et al., 2020). We created a text document for each post with a concatenation of text that consisted of its corresponding title, post, and comments. Several pre-processing steps were performed for these documents by implementing the Gensim (Rehurek and Sojka, 2010) and the Natural Language Toolkit Library (NLTK) (Bird et al., 2009).

**Figure 1:**
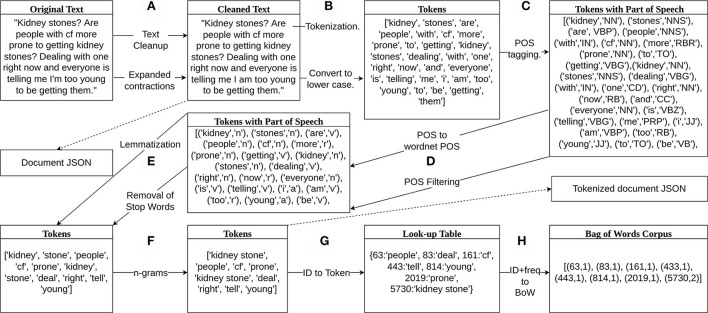
Data preprocessing diagram. **(A)** Removed web links, email addresses, and text within brackets and replaced contractions with expanded versions. **(B)** Tokenized and saved the cleaned text in lower case in a JSON file. **(C)** Part of speech (POS) tagging. **(D)** Converted POS to wordnet parts of speech and removed tokens if they are not an adjective, verb, noun, or adverb. **(E)** Lemmatized tokens and removed stop words. **(F)** Created bigrams and trigrams from the tokens and saved the list of remaining tokens in a JSON file. **(G)** Labeled tokens with an integer id and added them to a look-up table. **(H)** Represented bag of words corpus with pairs of two integers, the first integer is the token id from the look-up table and the second integer is the frequency of that token appearing in the document, e.g., token 5,730, “kidney stone” appears two times in the document and is represented by (5,730, 2).

1. We removed web links, email addresses, and text within brackets from the original text of each document. We also replaced contractions with their expanded versions, e.g., “can't” to “cannot,” “haven't,” to “have not.” An example of the original text and the cleaned version from this step for a single document can be seen in Figure 1A. Duplicate documents were removed to prevent unexpected bias in the LDA model. The cleaned documents were then saved as a JSON file.
2. We tokenized each cleaned document into tokens using the NLTK word tokenizer and converted the tokens to all lower case creating a list of tokens as shown in Figure 1B.
3. The list of tokens for each document was tagged with a part of speech (POS) using the POS-tagger module from NLTK. An example output of the tagging can be seen in Figure 1C.
4. We only included tokens tagged as adjective, adverb, noun, or adverb. The parts of speech were converted to wordnet parts of speech, e.g., all tags that start with N were converted to n for nouns. The tokens that were not tagged with the acceptable POS tag were removed as can be seen in Figure 1D.
5. Stop words were removed from the remaining list of tokens if they were found in the stop words in Gensim. Tokens were then lemmatized using the WordNetLemmatizer from NLTK. A lemma is the least inflected version of a word based on its meaning, e.g., as shown in Figure 1E.
6. Then, we created bigrams and trigrams from the tokens. Figure 1F shows the formation of the bigram “kidney stone” from the tokens “kidney” and “stone.” The list of remaining tokens for each document was then saved as a JSON file. The document and tokenized document files were used to reproduce topics for further model updates and enhancement.
7. A look-up table of integer ids and tokens was then created from the set of tokens such that each unique token is given an integer id. For example, from Figure 1G, the integer 63 is assigned to “people” and the integer 5,730 is assigned to “kidney stone.”
8. Finally, we generated corpora composed of pairs of token ids and token frequencies for each document. As an example, the list of tokens from the document in Figure 1F was combined with the look-up table in Figure 1G to create the bag of words corpus in Figure 1H. The first integer in each pair of numbers was the word id and the second integer was the number of times that token appears in that document.

### LDA model optimization

We created topic models using LDA and implemented through Gensim. Here, we employed hyperparameter tuning and LDA model optimization, with an aim of finding the most representative number of topics with the highest coherence automatically without further manual interpretation.

#### Hyperparameter tuning

There are many hyperparameters that are associated with the LDA model. These include a number of topics, chunksize (the number of documents to be used in each training chunk) (Hoffman et al., 2010), epochs (the maximum number of times the LDA algorithm is passed through the corpus when inferring the topic distribution) (Hoffman et al., 2010), alpha (*a priori* belief on the document to topic distribution) (Wallach et al., 2009), eta (the *a priori* belief on the topic to word distribution) (Wallach et al., 2009), offset (how much the first steps of the first iterations will be slowed down) (Hoffman et al., 2010), and decay (how quickly old information is forgotten) (Hoffman et al., 2010). With consideration of reports from the published studies (Wallach et al., 2009; Hoffman et al., 2010) and computational cost, we mainly tuned two main hyperparameters, the number of topics (with a range from 3 to 100) and the number of epochs (with a range from 1 to 100) through the corpus.

#### Optimization

To automate the procedure for determining the representative number of topics based on coherence, we investigated two different optimization algorithms in Hyperopt (Bergstra et al., 2013): adaptive Tree-Based Parzen Estimator (ATPE) (Wen et al., 2020) and random search (RS). ATPE uses an adaptive warm-up process to iteratively search for the best combination of hyperparameters in an automatic way (Wen et al., 2020). The algorithm adaptively adjusts the hyperparameter interval to guide the search. After this warm-up process, the algorithm uses a Tree-Based Parzen Estimator (TPE) to build a probability model of the objective function and uses it to select the most promising hyperparameters to evaluate the true objective function. (Bergstra et al., 2011) RS randomly chooses a number of topics and a number of epochs from a given range of values. The chosen number of topics and epochs were then used to generate an LDA model. The process was repeated for each trial. Since it was proved by Röder et al. (2015) that C_V_ as the coherence measure, which combines the cosine similarity with the normalized pointwise mutual information (NPMI) (Bouma, 2009), shows the strongest correlation with human ratings when compared to other measures, we applied C_V_ to assess the performance of the above two optimization algorithms.

### Top2Vec model generation

We evaluated the performance of the Top2Vec model with six different embedding methods that included doc2vec and five transformer-based pre-trained models. Doc2vec jointly learned embedded document and word vectors in the same semantic space using a distributed bag of words (DBOW) model. The DBOW model used document vectors to predict surrounding words in a context window in the document, which is similar to the word2vec skip-gram model. (Mikolov et al., 2013; Le and Mikolov, 2014) Thus, the word vectors with a shorter distance between the document vectors were more closely related to that document. Topic vectors were then calculated from the centroid or average of clusters of semantically similar document vectors (Angelov, 2020). Transformer-based models (Vaswani et al., 2017), such as BERT (Reimers and Gurevych, 2019, 2020) and Universal Sentence Encoders (USE) (Cer et al., 2018; Yang et al., 2019), take into account the context for each occurrence of a word. We evaluated five transformer-based pre-trained models, such as USE (Cer et al., 2018), multilingual USE (Yang et al., 2019), and 3 BERT models [all-MiniLM-L6-v2 (Reimers and Gurevych, 2019), distiluse-base-multilingual-cased (Reimers and Gurevych, 2020), and paraphrase-multilingual-MiniLM-L12-v2 (Reimers and Gurevych, 2020)]. The top topic words and their word scores for each topic were generated using Top2Vec. To be specific, the top words were the words that had semantic vectors with the smallest distance to the topic vector in semantic space. The scores for each word were the cosine similarity of the word vector to the topic vector. Similar to LDA, the top 10 words with the highest cosine similarity to each topic were applied to calculate the coherence for each topic and the mean coherence across all topics using Gensim.

## Results

In this experiment, we collected 9,176 posts and relevant comments from the subreddit of “r/CysticFibrosis.” Together, they formed 9,106 unique documents, which were tokenized into 11,028 tokens. The 11,028 tokens were applied to generate the token look-up table, which consisted of 7,903 unigrams, 2,921 bigrams, and 204 trigrams.

### LDA optimization results

**Figure 2:**
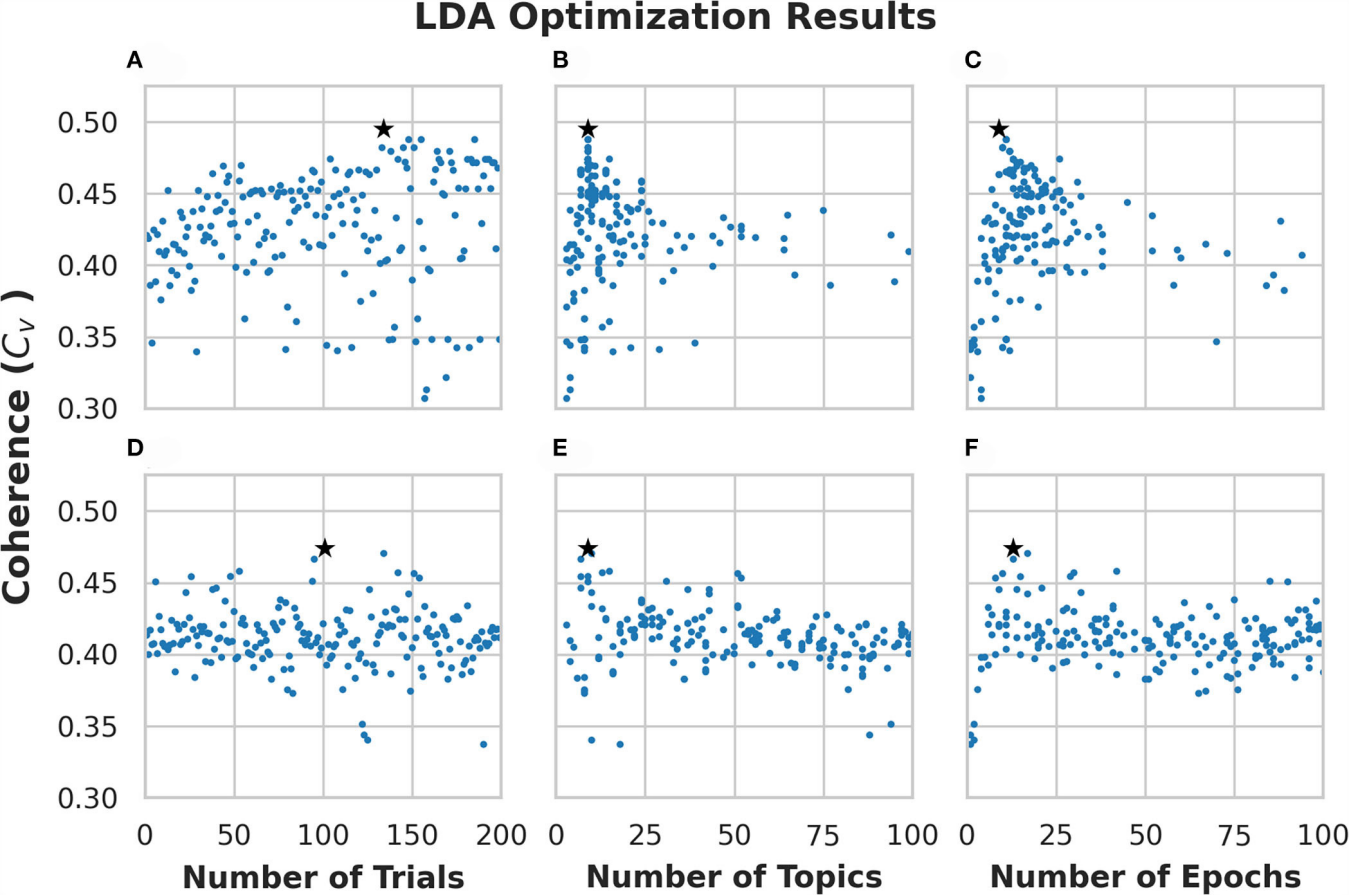
Optimization results for Latent Dirichlet Allocation (LDA) topic modeling of subreddit “r/CysticFibrosis.” **(A–C)** Show results from the 200 trials using adaptive Tree-Based Parzen Estimator (ATPE). **(D–F)** Show results from the 200 trials using a random search. Each dot represents a single model that was created for each trial. The stars denote the highest coherence model for the respective optimization method.

We ran 200 trials on each optimization algorithm (i.e., ATPE and RS). Each trial generates one model with different hyperparameters. Figures 2A–C shows the optimization results using ATPE, while Figures 2D–F shows the results using the RS algorithm corresponding to the numbers of trials/topics/epochs. Each dot in the figure corresponds to a generated model and thus, there are 200 models in each panel. The star in each figure denotes the trial with the highest coherence obtained with that algorithm. The model with the highest coherence using ATPE was generated with 9 topics and 9 passes on the trial of #134 with a coherence of 0.495, which is shown in Figures 2A–C. The model with the highest coherence based on the RS was generated with 9 topics and 13 passes on the trial of #101 with a coherence of 0.474, which is shown in Figures 2D–F.

**Figure 3:**
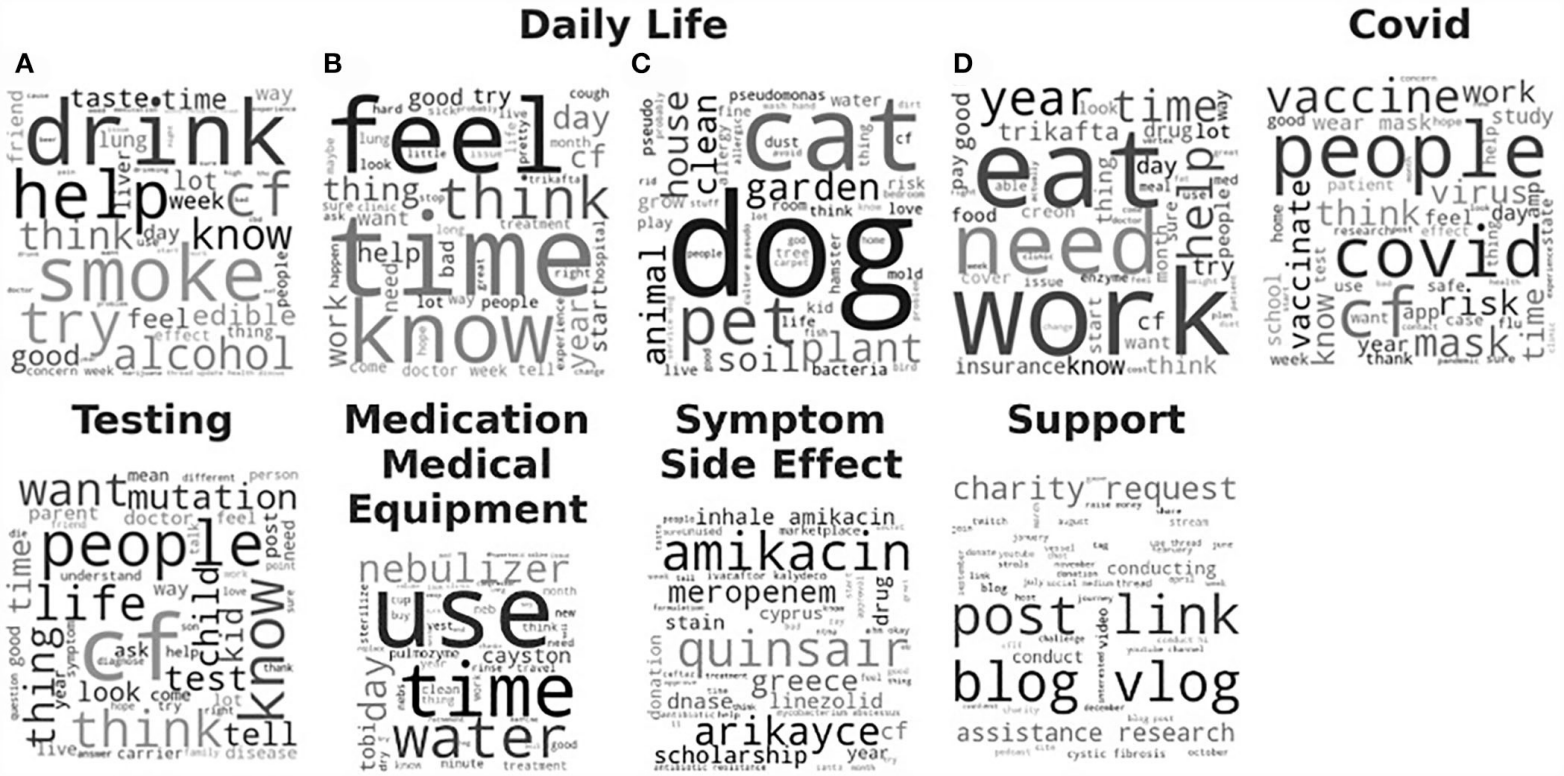
Word clouds for the 9 topics derived from the Latent Dirichlet Allocation (LDA) model with the highest coherence are sorted by category of interpreted topics. The font size of each word is relative to the probability of that word belonging to a document in that derived topic. Daily Life category consists of 4 subtopics: **(A)** Recreational drugs and alcohol use, **(B)** feeling, **(C)** pets, and **(D)** work and diet.

The LDA model with the highest coherence, the trial of #134 via ATPE, was then evaluated qualitatively based on the generated topics. Word clouds were created where the font size of each word corresponded to the relative probability of that word belonged to that topic as generated by the LDA model. We then grouped the topics into six topic categories, based on our manual interpretation, as “Daily Life,” “COVID,” “Testing,” “Support,” “Medication and Medical Equipment,” and “Symptom and Side Effect,” shown in Figure 3 and summarized in Table 1. The topic category of “Daily Life” consisted of four topics. (A) Recreational drugs and alcohol use, (B) feeling, (C) pets, and (D) work and diet. Obviously, those four topics are vague since the mixture words are included in each word cloud. Notably, a majority of documents, about 70%, were contributed to one of the four “Daily Life” topics. The topic about “Testing” was with 20% of the total documents. The topics about “COVID,” “Medication and Medical Equipment,” and “Support,” each was associated with <5% of the total documents, and the topic on “Symptom Side Effect” was with <0.1% of the documents. More detailed results can be found in Supplementary Table 1.

**Table 1:** Topic size and category names for the 9 derived topics from the Latent Dirichlet Allocation (LDA) model with the highest coherence.

	**Number**	**Number**	**Percentage**
**Topic category**	**topics**	**documents**	**documents**
Daily life	4	6,353	69.8
Testing	1	1,770	19.4
COVID	1	440	4.8
Medication and medical equipment	1	321	3.5
Support	1	211	2.3
Symptom and side effect	1	11	0.1

### Top2Vec results

**Figure 4:**
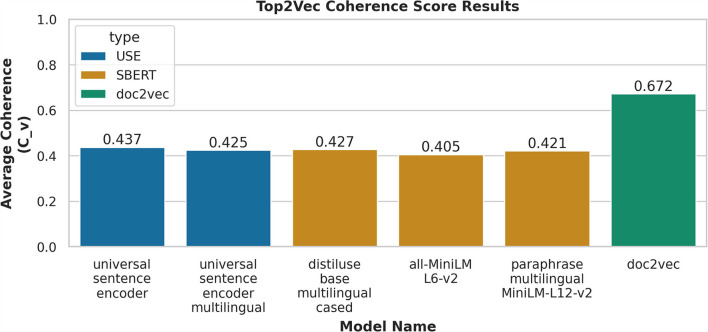
Top2Vec Results for the six embedding models evaluated on “r/CysticFibrosis”.

We examined multiple embedded models within Top2Vec, as shown in Figure 4, the doc2vec model resulted in the highest coherence of 0.672. The next score was 0.437 based on the transformer model of USE, which was about 23% less than doc2vec.

Top2vec with the doc2vec embedding model automatically generated a total of 68 topics. We grouped these 68 topics into eight categories as listed in Table 2 and found in more detail in Supplementary Table 2. The categories in order of the number of documents were “Medication and Medical Equipment” with 20.0%, “Daily Life” with 19.4%, “Symptom and Side Effect” with 17.3%, “Support” with 15.2%, “Health” with 12.7%, “Health care” with 7.4%, “Testing” with 4.5%, and “COVID” with 3.4%.

**Table 2:** Topic size and category names for the 68 derived topics using Top2Vec with doc2vec embedding model.

**Topic**	**Number**	**Number**	**Percentage**
**category**	**topics**	**documents**	**documents**
Medication and medical equipment	14	1,824	20.0
Daily life	12	1,770	19.4
Symptom and side effect	15	1,579	17.3
Support	7	1,384	15.2
Health	11	1,158	12.7
Healthcare	3	671	7.4
Testing	3	407	4.5
COVID	3	313	3.4

**Figure 5:**
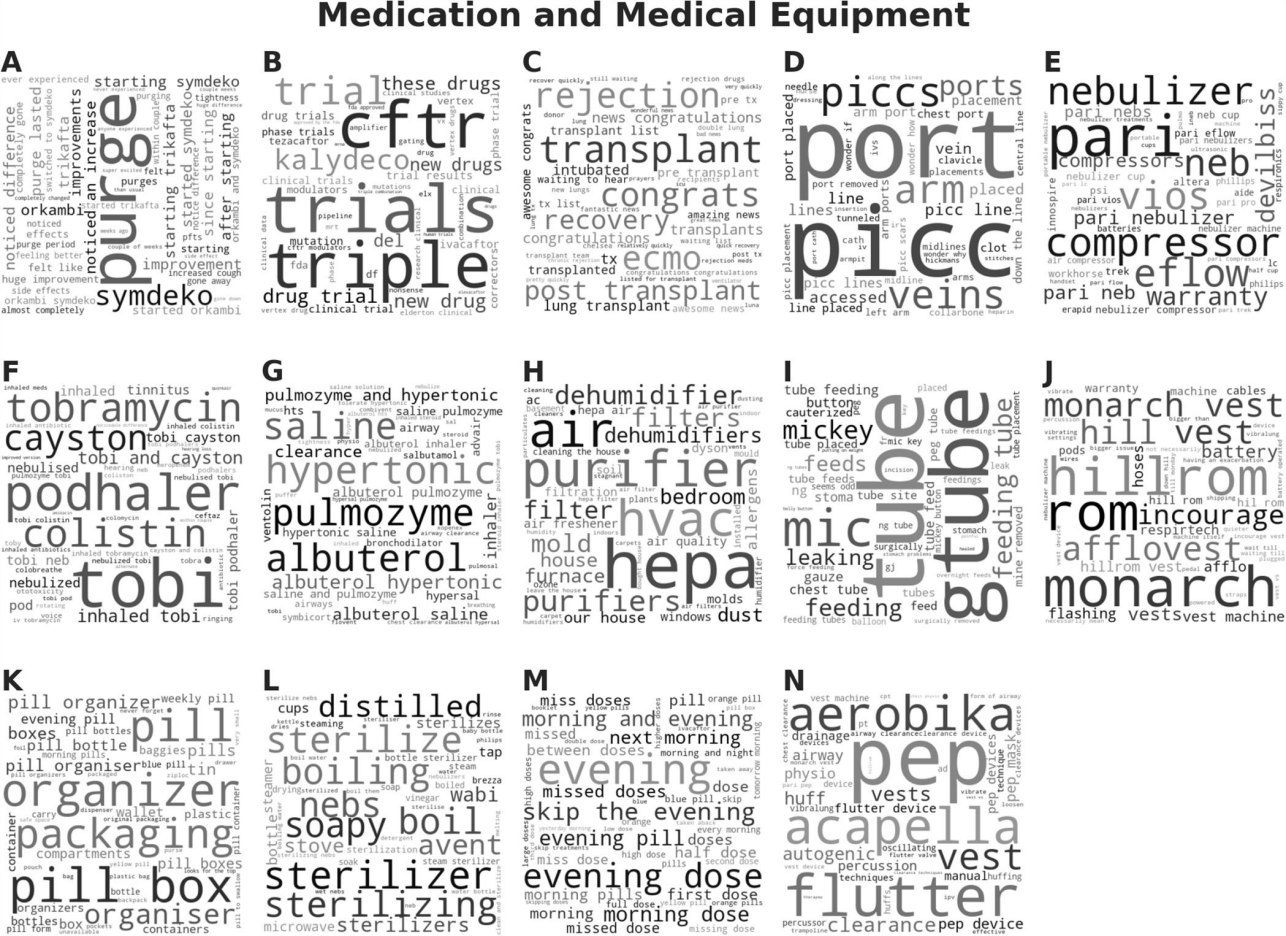
Word clouds for the topics that were grouped into the medication and medical equipment category for the Top2Vec model using doc2vec embedding. Topics related to “Medication and Medical Equipment” in include: **(A)** oral drug use, **(B)** drug trials, **(C)** transplantation, **(D)** intravenous medication, **(E)** nebulizers, **(F)** inhaled antibiotics, **(G)** inhaled drugs, **(H)** air quality devices, **(I)** feeding tubes, **(J)** high-frequency chest wall oscillators, **(K)** pill organization, **(L)** sterilization, **(M)** medication dosing, and **(N)** airway clearing devices, which are main drug category and medical equipment CF patients use and care about.

**Figure 6:**
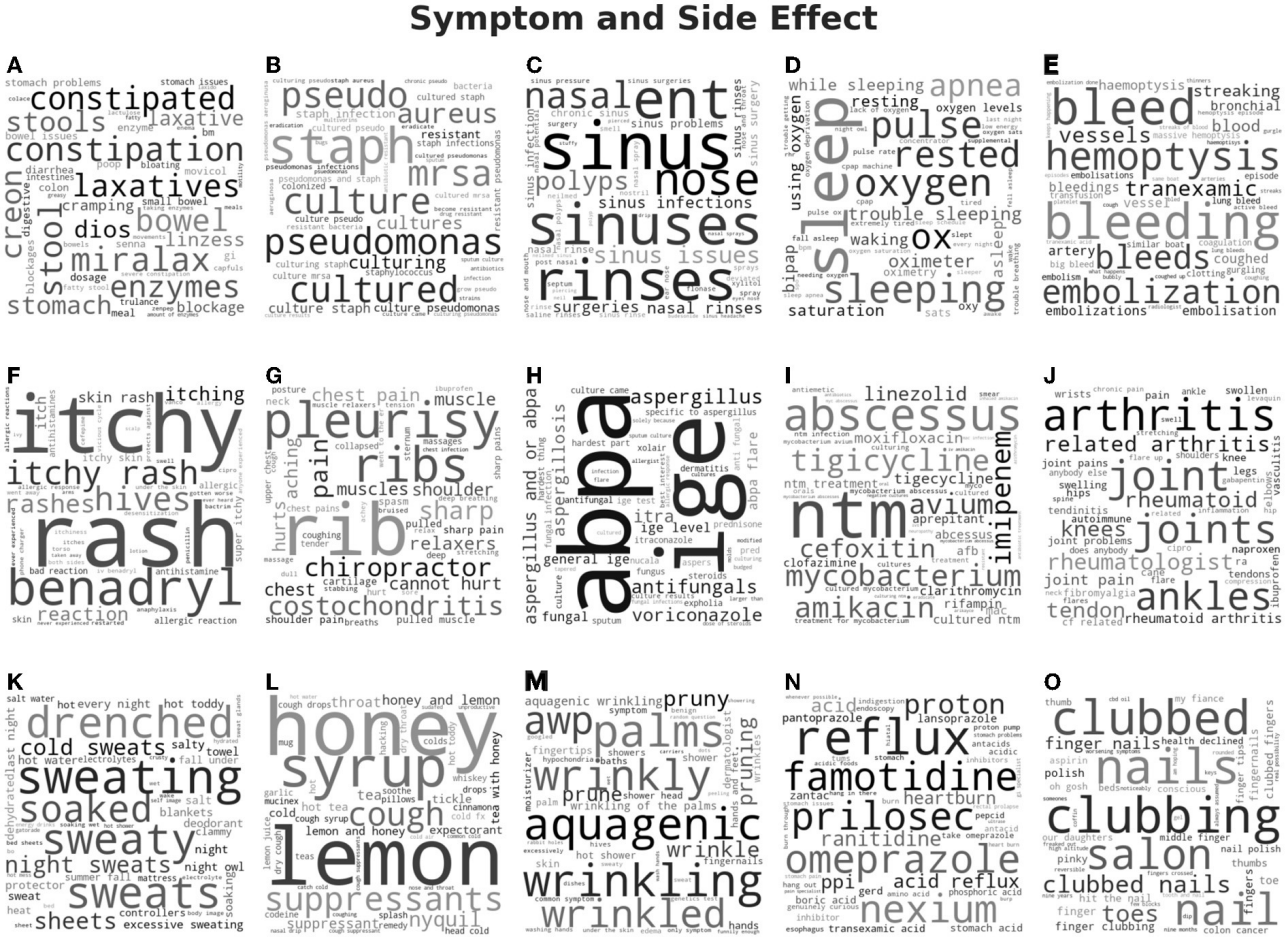
Word clouds for the topics that were grouped into the symptom and side effect category for the Top2Vec model using doc2vec embedding.

Word clouds were generated with the font size of each word corresponding to the cosine similarity score, which measures how close the word vector was to the topic vector in semantic space. Thus, words with a high score were located at a small distance away from the topic vector in semantic space and were semantically closely related. Word clouds for the topics related to the topic categories of “Medication and Medical Equipment” are shown in Figure 5 and “Symptom and Side Effect” are shown in Figure 6. The word clouds for the topics related to “Daily Life,” “Support,” “Health,” “Health care,” “Testing,” and “COVID” can be found in the Supplementary material in Supplementary Figures 1–6. The topics related to “Medication and Medical Equipment” in Figure 5 include (A) oral drug use, (B) drug trials, (C) transplantation, (D) intravenous medication, (E) nebulizers, (F) inhaled antibiotics, (G) inhaled drugs, (H) air quality devices, (I) feeding tubes, (J) high-frequency chest wall oscillators, (K) pill organization, (L) sterilization, (M) medication dosing, and (N) airway clearing devices, which are main drug category and medical equipment CF patients use and care about. The topics related to “Symptom and Side Effect” in Figure 6 included (A) constipation, (B) bacterial infections, (C) sinus infections, (D) sleep issues, (E) bleeding, (F) rashes, (G) chest and abdominal pain, (H) fungal infections, (I) bacterial infection medication, (J) joint pain, (K) sweating, (L) cough remedies, (M) wrinkled palms, (N) acid reflux, and (O) clubbed fingers, which are corresponding to some of the main symptoms observed among CF patients^5^.

## Discussions

In this study, we explored LDA and Top2Vec, two widely applied topic modeling algorithms on Reddit with an overarching aim of uncovering the needs/concerns patients with CF might have. From this study, we concluded that Top2Vec with the embedded algorithm of doc2vec outperforms LDA, not only because Top2Vec automates the process of topic modeling without pre-determining several hyperparameters, e.g., the number of topics, but also because it generates more concrete topics, as shown in the Results section.

As outlined in Angelov (2020), Top2Vec and probabilistic generative models, such as the LDA, differ in how they model a topic. LDA models topics as distributions of words and these distributions are used to recreate the original document word distributions (Blei et al., 2003). In contrast, Top2Vec utilizes a semantic space (Griffiths et al., 2007) where the distance between vectors represents the semantic association. Top2Vec uses topic, document, and word vectors where the distance between the vectors represents semantic similarity. Semantically similar document vectors will be clustered near the word vectors that best describe them. The centroid or average of those document vectors in a cluster is calculated and forms the topic vector. Because of the assumption that the number of clusters of document vectors equals the number of topics, *a priori* knowledge of the number of topics is not required. This is a major advantage over the LDA. Additionally, since it uses semantic space to represent words, there is no need to do pre-processing to remove stop words or perform lemmatization or stemming. Because only semantically similar words are found near the documents, the uninformative stop words are not found near the document vectors. As a preliminary study, we found that Top2Vec discovered topics that were more informative and representative of the subreddit.

Doc2vec embedding algorithm implements the DBOW model to predict surrounding words in a context window in the document, but with the limitation of using the same vector for words appearing in different contexts. (Le and Mikolov, 2014) Since transformer-based models take into account the context for each occurrence of a word, we were expecting they would perform better (Vaswani et al., 2017). However, this experiment demonstrated that the doc2vec model could generate more coherent models on social media data, which have limited content in general. We speculate that this may be due to the difference in the text used to pre-train the models as compared to the text found on Reddit. To further prove our findings, we proposed to expand our experiment to other rare disease-related subreddits, which will be described in a separate manuscript.

In this study, Top2Vec programmatically generated 68 topics corresponding to eight different categories, which overlap with the main topic categories from LDA. However, by looking at those topic categories in the word cloud, which are shown in Figures 3, 5, 6. Obviously more coherent and concrete textual information has been applied for topic modeling by Top2Vec. For instance, the topics included in the topic category of “Symptom and Side Effect” are more meaningful and deliver more interesting results, which can directly help us to access the main symptoms and/or side effects that patients with CF are suffering, i.e., (A) constipation, (B) bacterial infections, (C) sinus infections, (D) sleep issues, (E) bleeding, (F) rashes, (G) chest and abdominal pain, (H) fungal infections, (I) bacterial infection medication, (J) joint pain, (K) sweating, (L) cough remedies, (M) wrinkled palms, (N) acid reflux, and (O) clubbed fingers. Some of them are primary symptoms associated with CF according to the Human Phenotype Ontology^6^, others might be side effects, which may also be worthy to investigate further. The reason leading to the discrepancy in topic modeling results between LDA and Top2Vec, besides the underneath mechanisms they relied on, might be the uneven number of documents that were applied by LDA for topic modeling. In total, 70% of the documents were used by LDA to generate the topic category of “Daily Life,” where the associated topics are very vague, whereas only 0.1% of the documents were for “Symptom and Side Effect” with very less useful information.

As a proof-of-concept, we will further extend this work by analyzing more rare disease-related subreddits with an aim of accessing urgent needs for rare disease patients from social media and consequently identifying research gaps and initiating new research activities.

## Data availability statement

The original contributions presented in the study are included in the article/Supplementary material, further inquiries can be directed to the corresponding authors.

## Author contributions

BK conducted data analysis and drafted the manuscript. SQ managed the project, participated in the discussion, and helped on the manuscript. YX participated in the discussion. QZ conceived and supervised the project and drafted the manuscript. All authors contributed to the article and approved the submitted version.

## Conflict of interest

The authors declare that the research was conducted in the absence of any commercial or financial relationships that could be construed as a potential conflict of interest.

## Publisher's note

All claims expressed in this article are solely those of the authors and do not necessarily represent those of their affiliated organizations, or those of the publisher, the editors and the reviewers. Any product that may be evaluated in this article, or claim that may be made by its manufacturer, is not guaranteed or endorsed by the publisher.

## Footnotes

^1^Un resolution. *Un Resolution on Persons Living With a Rare Disease*.

^2^Foundation CF. *About Cystic Fibrosis*. Available online at: https://www.cff.org/What-is-CF/About-Cystic-Fibrosis.

^3^CysticFibrosis. *CysticFibrosis.com, How Common is Cystic Fibrosis?* Available online at: https://cysticfibrosis.com/statistics.

^4^Podolak, M. D. *T, mattpodolak/pmaw: A multithread Pushshift.io API Wrapper for reddit.com comment and submission searches*. Available online at: https://github.com/mattpodolak/pmaw.

^5^Cystic-Fibrosis.com. *Cystic-Fibrosis.com, Clubbed Fingers and Toes*. Available online at: https://cystic-fibrosis.com/symptoms/clubbing-fingers-toes.

^6^Ontology, H. P. *HPO associations for Cystic Fibrosis*. Available online at: https://hpo.jax.org/app/browse/disease/OMIM:219700.
