# Supplementary material for: Experiments with LDA and Top2Vec for embedded topic discovery on social media data—A case study of cystic fibrosis
Source: Front Artif Intell. 2022 Aug 18;5:948313. doi: 10.3389/frai.2022.948313 (PMC9433987; doi:10.3389/frai.2022.948313)
Supplement: Supplementary file 1 [file Data_Sheet_1.PDF]

Experiments with LDA and Top2Vec for Embedded Topic Discovery on Social Media Data - A Case Study of Cystic Fibrosis

Topic Number	Topic Sizes	Coherence	Top 10 words
1	512	0.420	'drink', 'smoke', 'help', 'cf', 'try', 'alcohol', 'know', 'think', 'good', 'edible'
2	4378	0.337	'time', 'feel', 'know', 'think', 'thing', 'day', 'cf', 'year', 'work', 'help'
3	211	0.718	'post link blog vlog', 'charity request assistance research', 'conducting', 'conduct', 'video', 'cystic fibrosis', 'stream', 'blog', 'weekly thread use thread', 'weekly october use thread'
4	440	0.544	'people', 'covid', 'cf', 'vaccine', 'mask', 'think', 'risk', 'virus', 'vaccinate', 'time'
5	1770	0.379	'cf', 'people', 'know', 'think', 'life', 'want', 'thing', 'mutation', 'test', 'child'
6	321	0.568	'use', 'time', 'water', 'nebulizer', 'day', 'cayston', 'tobi', 'pulmozyme', 'clean', 'minute'
7	45	0.721	'dog', 'cat', 'pet', 'plant', 'garden', 'house', 'soil', 'animal', 'clean', 'grow'
8	1418	0.333	'work', 'eat', 'need', 'year', 'help', 'time', 'trikafta', 'know', 'think', 'cf'
9	11	0.433	'amikacin', 'quinsair', 'arikayce', 'meropenem', 'greece', 'scholarship', 'cf', 'inhale amikacin', 'dnase', 'linezolid'

Supplementary Table 1. Table of generated topics for highest coherence LDA model. Number of documents, coherence (C_V) per topic, and top 10 words with the highest probability of belonging to that topic.

Topic Number	Topic Sizes	Coherence	Top 10 words

Supplementary Material

1	422	0.388	'meme', 'funny enough', 'haha yeah', 'oh man', 'funny', 'funny story', 'hey man', 'jokes', 'oh okay', 'humor'
2	320	0.510	'relationship', 'dating', 'relationships', 'married', 'boyfriend', 'partner', 'life', 'friends', 'person', 'dating someone'
3	309	0.729	'constipation', 'miralax', 'stool', 'constipated', 'enzymes', 'laxatives', 'bowel', 'creon', 'stools', 'stomach'
4	302	0.614	'donate', 'fundraising', 'donations', 'foundation', 'awareness', 'cff', 'donated', 'donation', 'fibrosis awareness', 'cf foundation'
5	294	0.309	'weekly checkup', 'going and discuss', 'checkup', 'discuss any', 'any concerns', 'update everyone', 'concerns', 'discuss', 'during the week', 'update'
6	290	0.476	'purge', 'symdeko', 'starting symdeko', 'orkambi', 'starting trikafta', 'since starting', 'improvement', 'after starting', 'purge lasted', 'trikafta'
7	288	0.902	'insurance', 'compass', 'copay', 'coverage', 'medicaid', 'deductible', 'insurance plan', 'copays', 'pay', 'healthwell'
8	271	0.640	'trials', 'cftr', 'triple', 'trial', 'kalydeco', 'drug trial', 'new drug', 'these drugs', 'new drugs', 'del'
9	260	0.284	'are conducting', 'blog', 'vlog', 'conducting', 'vlog calls', 'post links', 'blog post', 'charity and requests', 'requests', 'links to your'
10	211	0.705	'test', 'sweat', 'testing', 'genetic', 'genetic test', 'diagnosis', 'mutations', 'sequencing', 'genetic tests', 'sweat test'
11	207	0.760	'canada', 'vertex', 'access', 'government', 'price', 'provincial', 'provinces', 'negotiations', 'approval', 'approved in canada'
12	201	0.397	'cystic fibrosis', 'fibrosis', 'cystic', 'wide open', 'group cystic', 'fibrosis awareness', 'disease causing', 'genetic disease', 'little boy', 'genetic diseases'
13	192	0.854	'butter', 'peanut', 'cheese', 'avocado', 'fat', 'calories', 'milk', 'eat', 'yogurt', 'olive'
14	185	0.569	'transplant', 'rejection', 'congrats', 'ecmo', 'post transplant', 'recovery', 'news congratulations', 'congratulations', 'intubated', 'tx'
15	176	0.316	'denver', 'consider moving', 'city', 'major city', 'jacksonville', 'tampa', 'moved away', 'florida', 'center', 'centers'
16	169	0.443	'darkjarris', 'promo', 'promotional', 'vlogs', 'blogs', 'moderators', 'mod', 'cysticfibrosis', 'moderator', 'rule of the sub'
17	169	0.753	'covid', 'vaccinated', 'distancing', 'virus', 'covid pandemic', 'unvaccinated', 'pandemic', 'vaccine', 'precautions', 'stay safe'
18	167	0.739	'staph', 'pseudomonas', 'mrsa', 'pseudo', 'cultured', 'culture', 'aureus', 'culturing', 'cultures', 'culture staph'
19	152	0.792	'port', 'picc', 'piccs', 'veins', 'arm', 'ports', 'picc line', 'accessed', 'line', 'lines'
20	150	0.928	'weed', 'edibles', 'smoking', 'vaping', 'marijuana', 'smoke', 'smoking weed', 'thc', 'smoked', 'vape'
21	149	0.626	'games', 'discord', 'gaming', 'gamer', 'play games', 'gamers', 'play', 'server', 'minecraft', 'xbox'
22	146	0.808	'sinus', 'sinuses', 'rinses', 'ent', 'nose', 'nasal', 'polyps', 'sinus issues', 'surgeries', 'sinus

			infections'
23	144	0.467	'sleep', 'sleeping', 'oxygen', 'pulse', 'rested', 'ox', 'apnea', 'trouble sleeping', 'oximeter', 'while sleeping'
24	137	0.714	'weight', 'lbs', 'gain', 'gained', 'skinny', 'gaining', 'calories', 'pounds', 'lose weight', 'gain lbs'
25	133	0.768	'depression', 'anxiety', 'psychiatrist', 'antidepressants', 'mental', 'prozac', 'zoloft', 'depression anxiety', 'anxiety depression', 'anxiety and depression'
26	133	0.863	'bleeding', 'bleed', 'hemoptysis', 'embolization', 'bleeds', 'tranexamic', 'vessels', 'streaking', 'blood', 'haemoptysis'
27	125	0.766	'movie', 'apart movie', 'characters', 'romance', 'watch movie', 'seen the movie', 'character', 'plot', 'actors', 'hollywood'
28	124	0.899	'pari', 'compressor', 'eflow', 'vios', 'nebulizer', 'neb', 'warranty', 'devilbiss', 'pari nebulizer', 'pari neb'
29	123	0.585	'survey', 'participation', 'link to the survey', 'participate', 'online survey', 'participants', 'research project', 'anonymous', 'research study', 'compensation'
30	122	0.931	'sugars', 'insulin', 'cfrd', 'glucose', 'sugar', 'cgm', 'diabetic', 'diabetes', 'dexcom', 'blood sugar'
31	121	0.688	'cardio', 'running', 'workout', 'gym', 'bike', 'exercises', 'training', 'weights', 'exercise', 'treadmill'
32	116	0.668	'job', 'jobs', 'great job', 'boss', 'employers', 'employment', 'employer', 'career', 'office job', 'employees'
33	109	0.848	'tobi', 'podhaler', 'tobramycin', 'colistin', 'cayston', 'tobi and cayston', 'inhaled tobi', 'tobi cayston', 'nebulized', 'pod'
34	106	0.803	'rash', 'itchy', 'benadryl', 'itchy rash', 'hives', 'rashes', 'itching', 'reaction', 'skin rash', 'itch'
35	106	0.769	'hypertonic', 'albuterol', 'pulmozyme', 'saline', 'albuterol hypertonic', 'pulmozyme and hypertonic', 'inhaler', 'albuterol saline', 'clearance', 'albuterol pulmozyme'
36	103	0.554	'daughter', 'born', 'old daughter', 'our daughter', 'your daughter', 'parent', 'overwhelming', 'advancements', 'meconium', 'parents'
37	99	0.779	'hepa', 'purifier', 'hvac', 'air', 'purifiers', 'dehumidifier', 'filters', 'mold', 'filter', 'dehumidifiers'
38	98	0.632	'tattoo', 'tattoos', 'artist', 'tats', 'tattoo artist', 'roses', 'tattooed', 'rose', 'artists', 'ink'
39	98	0.489	'condolences', 'loved', 'grieve', 'wyatt', 'loss', 'peace', 'best friend', 'joy', 'loved ones', 'wishes'
40	96	0.598	'hair', 'biotin', 'acne', 'scalp', 'dermatologist', 'vitamin', 'bald', 'iron', 'testosterone', 'vitamins'
41	96	0.511	'tube', 'gtube', 'mic', 'mickey', 'feeding tube', 'feeding', 'leaking', 'feeds', 'tube feeding', 'button'
42	93	0.790	'monarch', 'hillrom', 'rom', 'monarch vest', 'vest', 'afflovest', 'hill', 'incourage', 'battery', 'vests'
43	93	0.727	'spirometer', 'nuvoair', 'numbers', 'spirometry', 'spirometers', 'pft', 'nuvo', 'fev', 'accurate', 'microlife'

44	92	0.668	'rib', 'pleurisy', 'ribs', 'costochondritis', 'chiropractor', 'sharp', 'pain', 'chest pain', 'relaxers', 'chest'
45	90	0.906	'sperm', 'vas', 'semen', 'ivf', 'deferens', 'infertile', 'fertility', 'ejaculate', 'males', 'sperm count'
46	89	0.761	'abpa', 'ige', 'aspergillus', 'antifungals', 'voriconazole', 'aspergillus and or abpa', 'itra', 'aspergillosis', 'general ige', 'ige level'
47	89	0.473	'pill box', 'organizer', 'pill', 'packaging', 'organiser', 'pill organizer', 'pill organiser', 'boxes', 'pills', 'tin'
48	86	0.577	'carrier', 'child', 'carriers', 'adoption', 'chance', 'ivf', 'pgd', 'second chance', 'amnio', 'abortion'
49	82	0.798	'sterilizer', 'sterilize', 'boil', 'boiling', 'sterilizing', 'nebs', 'soapy', 'distilled', 'avent', 'sterilizers'
50	79	0.919	'flight', 'tsa', 'plane', 'luggage', 'airlines', 'airline', 'attendant', 'airport', 'flying', 'fly'
51	79	0.559	'song', 'sing', 'singing', 'singer', 'lyrics', 'songs', 'music', 'band', 'album', 'playlist'
52	77	0.910	'cats', 'dogs', 'cat', 'pet', 'dog', 'pets', 'dander', 'animals', 'birds', 'litter'
53	74	0.902	'pfizer', 'vaccine', 'moderna', 'jab', 'booster', 'az', 'second shot', 'pfizer vaccine', 'vaccines', 'shot'
54	73	0.780	'ntm', 'abscessus', 'mycobacterium', 'tigicycline', 'amikacin', 'imipenem', 'avium', 'cefoxitin', 'linezolid', 'tigecycline'
55	71	0.738	'arthritis', 'joint', 'joints', 'ankles', 'rheumatologist', 'related arthritis', 'rheumatoid', 'knees', 'tendon', 'joint pain'
56	70	0.689	'masks', 'cambridge', 'vogmask', 'mask', 'vogmasks', 'reusable', 'surgical', 'vog', 'disposable', 'wearer'
57	67	0.495	'kumar', 'usha', 'ovarian', 'cysts', 'ovarian cysts', 'cyst', 'dr usha', 'stage lymphoma', 'scheme of things', 'testicles'
58	65	0.561	'sweating', 'sweats', 'drenched', 'sweaty', 'soaked', 'night sweats', 'sheets', 'cold sweats', 'excessive sweating', 'every night'
59	65	0.517	'evening', 'evening dose', 'skip the evening', 'morning and evening', 'evening pill', 'morning dose', 'next morning', 'missed doses', 'morning pills', 'dose'
60	64	0.743	'pregnant', 'pregnancy', 'pregnancies', 'weeks pregnant', 'getting pregnant', 'while pregnant', 'months pregnant', 'got pregnant', 'birth', 'pregnant women'
61	63	0.774	'pep', 'flutter', 'acapella', 'aerobika', 'vest', 'clearance', 'vests', 'autogenic', 'huff', 'pep device'
62	62	0.612	'lemon', 'honey', 'syrup', 'suppressants', 'cough', 'nyquil', 'suppressant', 'tea', 'throat', 'honey and lemon'
63	57	0.855	'drunk', 'hangovers', 'drinking', 'alcohol', 'drinking alcohol', 'drink alcohol', 'beers', 'beer', 'liquor', 'drank'
64	50	0.579	'sex', 'during sex', 'sex drive', 'sexual', 'pelvic', 'horny', 'orgasm', 'opposite sex', 'intercourse', 'lube'
65	47	0.659	'wrinkling', 'palms', 'aquagenic', 'wrinkly', 'awp', 'wrinkled', 'pruny', 'wrinkle', 'pruning', 'prune'

66	42	0.894	'reflux', 'famotidine', 'omeprazole', 'nexium', 'prilosec', 'proton', 'ranitidine', 'acid reflux', 'ppi', 'acid'
67	35	0.565	'app', 'apps', 'android', 'app called', 'app store', 'download', 'ios', 'tracking', 'developer', 'track'
68	33	0.370	'clubbing', 'nails', 'clubbed', 'nail', 'salon', 'clubbed nails', 'toes', 'finger nails', 'finger clubbing', 'clubbed fingers'

Supplementary Table 2. Table of generated topics for Top2Vec with doc2vec embedding model.

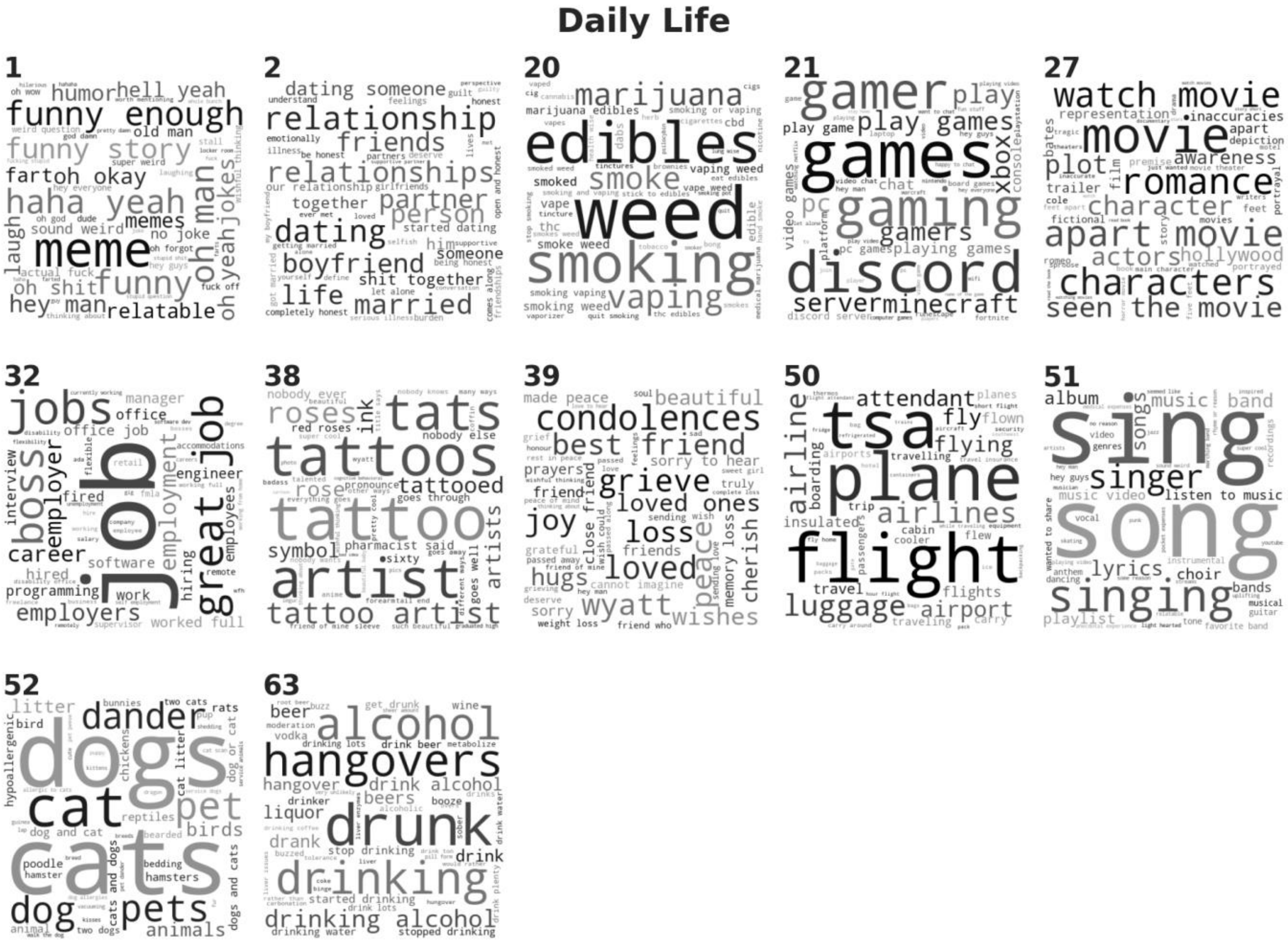

Supplementary Figure 1. Top2Vec with doc2vec embedding word clouds for the daily life category.

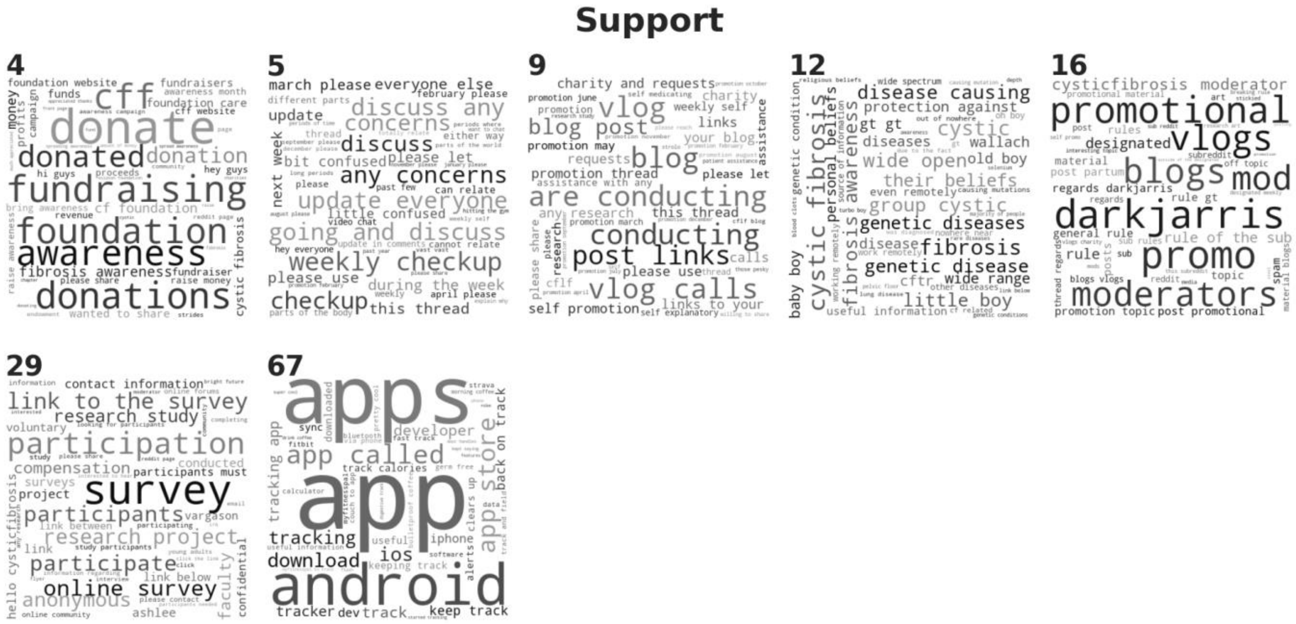

Supplementary Figure 2. Top2Vec with doc2vec embedding word clouds for the support category.

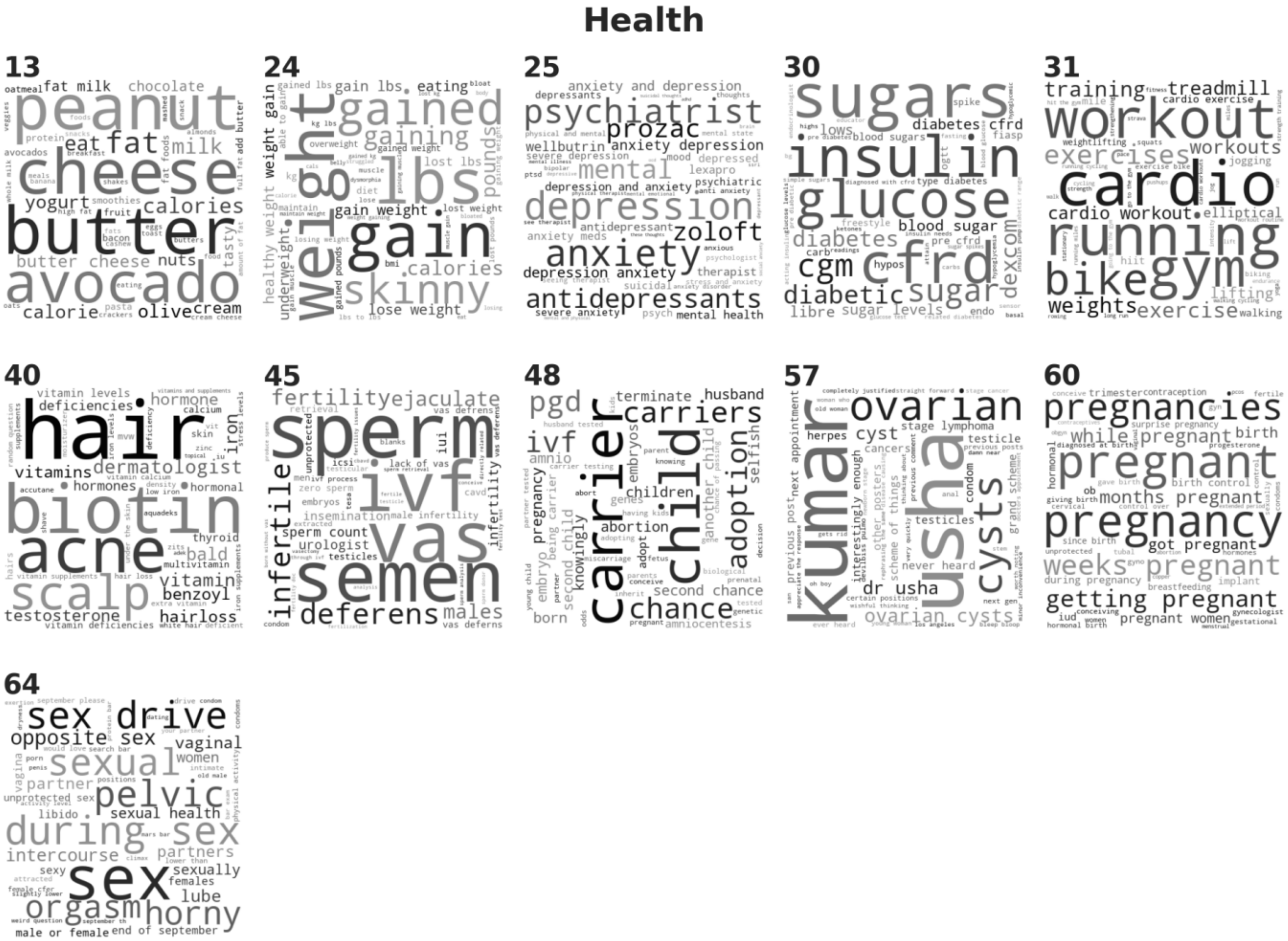

Supplementary Figure 3. Top2Vec with doc2vec embedding word clouds for the health category.

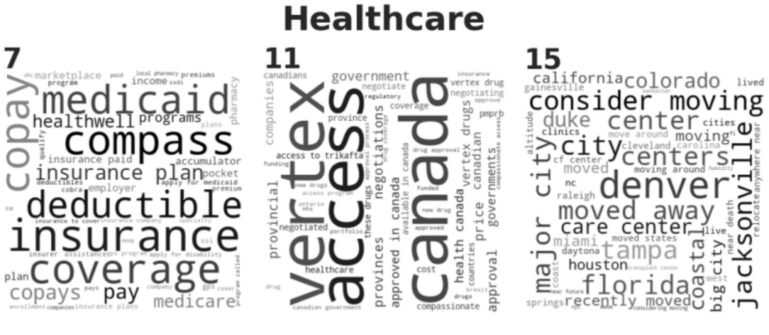

Supplementary Figure 4. Top2Vec with doc2vec embedding word clouds for the healthcare category.

Supplementary Material

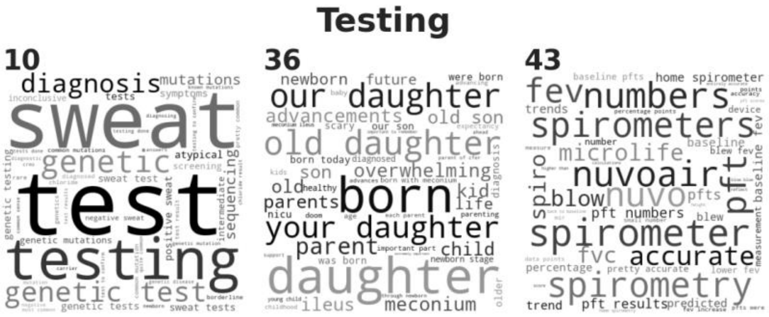

Supplementary Figure S5. Top2Vec with doc2vec embedding word clouds for the testing category.

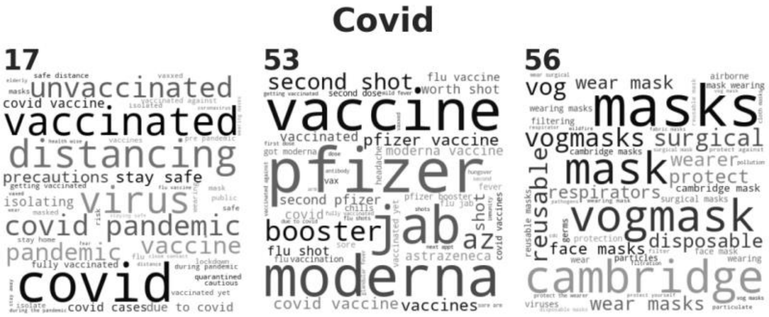

Supplementary Figure 6. Top2Vec with doc2vec embedding word clouds for the Covid category.
